# Effects of Qingshen Granules on Immune Function in Patients with Comorbid Chronic Renal Failure and Damp-Heat Syndrome: A Multicenter, Randomized, Controlled Trial

**DOI:** 10.1155/2020/5057894

**Published:** 2020-10-12

**Authors:** Dong Wang, Yiping Wang, Chuanping Li, Shifu Liu, Lei Zhang, Hua Jin

**Affiliations:** ^1^Department of Nephrology, The First Affiliated Hospital of Anhui University of Traditional Chinese Medicine, Hefei 230031, China; ^2^Department of Nephrology, Lu'an Hospital of Traditional Chinese Medicine, Lu'an 237006, China; ^3^Department of Nephrology, Wuhu Hospital of Traditional Chinese Medicine, Wuhu 241000, China

## Abstract

**Objective:**

The current study sought to compare the effects of the addition of Qingshen granules to conventional Western medicine on immune function in patients with comorbid chronic renal failure and damp-heat syndrome and to explore the possible mechanisms responsible for any differences observed.

**Methods:**

Through a multicenter, randomized, controlled study, a total of 282 eligible patients were divided into experimental (*n* = 136) and control groups (*n* = 146). All of the patients were treated with conventional Western medical therapy. The experimental group also received Qingshen granules three times daily for 12 weeks. Clinical efficacy was observed in the two groups. Peripheral blood levels of CD4^+^ T cells, CD8^+^ T cells, Th17 cells, nuclear factor-*κ*B p65 (NF-*κ*B p65) activity, serum interleukin-17 (IL-17), serum interleukin-6 (IL-6), tumor necrosis factor-*α* (TNF-*α*), tumor necrosis factor receptor-associated factor 6 (TRAF6), fibronectin (FN), and type IV collagen (Col-IV) were detected in both groups.

**Results:**

The total clinical curative effective rate was significantly higher (*p* < 0.05) in the experimental group (79.41%) than in the control group (67.12%). Before treatment, there were no significant differences in CD4^+^/CD8^+^ T cell ratio, Th17 cell level, NF-*κ*B p65 activity, serum IL-17, IL-6, TNF-*α*, TRAF6, FN, and Col-IV between the experimental and control groups (*p* > 0.05); however, all of the measures were significantly higher than those observed in a healthy comparison group (*p* < 0.05 or *p* < 0.01). After treatment, the above indexes in the experimental group were significantly lower than those before treatment (*p* < 0.05 or *p* < 0.01). Similarly, NF-*κ*B p65 activity, serum IL-17, TNF-*α*, TRAF6, FN, and Col-IV in the control group were significantly lower than the levels observed prior to treatment (*p* < 0.05 or *p* < 0.01); however, while all of the other indexes were lower than those observed before treatment, the differences were not statistically significant (*p* > 0.05).

**Conclusion:**

Qingshen granules adjust immune dysfunction, improve immunity mediated inflammatory response, and attenuate renal fibrosis in patients with comorbid chronic renal failure and damp-heat syndrome.

## 1. Preface

With the continual improvement in the standard of living, the quickening of the overall pace of life, and the increase of environmental pollution, the incidence rate of chronic kidney disease (CKD) has been increasing annually. In 2017, Wang et al. [1] conducted a meta-analysis of 28 CKD screening studies in China and found that the prevalence rate in adults was 13.39%. Based on this, it is estimated that the number of CKD cases in China will reach 190 million, seriously endangering life and health. Chronic renal failure (CRF) is a serious stage in the development of various forms of CKD, which eventually progresses to end-stage renal disease (ESRD). This disease requires alternative treatments to maintain life. Alone or combined use of traditional Chinese medicine in the treatment of CRF shows some advantages, but there are few reports of multicenter, randomized, and controlled clinical studies with large sample sizes. Damp-heat syndrome is the primary presentation of CRF, with blood stasis occurring through the course of the disease; heat clearing, dampness removal, and stasis removal are several of the primary treatment methods used for CRF [2]. Based on this, our hospital has developed a Qingshen granule, which has the effect of clearing away heat, dampness, and blood stasis. Continuing research examining the properties of Qingshen granules has demonstrated their importance in traditional Chinese medicine (TCM).

Qingshen granules are prepared in our hospital. They have been used in clinical practice for nearly 20 years. Previous research [3–6] has shown that they can obviously improve clinical symptoms in CRF patients with damp-heat syndrome, protect renal function, and delay the progress of CRF. In this study, 282 patients with comorbid CRF and damp-heat syndrome from the First Affiliated Hospital of Anhui University of Traditional Chinese Medicine, Lu'an Hospital of Traditional Chinese Medicine, and Wuhu Hospital of Traditional Chinese Medicine were included. It was found in [7] that the experimental protocol could significantly improve the clinical symptoms of CRF patients with damp-heat syndrome, reduce the integral value of TCM syndrome, reduce 24-hour urine protein quantity, and lower serum creatinine level; however, the specific mechanism responsible for these effects remains to be elucidated. The current study sought to investigate the effects of Qingshen granules on patients with comorbid CRF and damp-heat syndrome from the perspective of immune inflammation and to explore the possible underlying mechanisms.

## 2. Data and Methods

### 2.1. Diagnostic Criteria of Western Medicine

The current study utilized the CKD diagnosis and staging criteria proposed in the Kidney Disease: Improving Global Outcomes (KDIGO) guidelines proposed in 2009 [8]. The diagnostic criteria of CKD were (1) renal damage (i.e., abnormal renal structure or function) ≥3 months, with or without decreased glomerular filtration rate (GFR), which can present with abnormal pathological exam, positive renal function damage index (e.g., blood or urine composition abnormality), or abnormal imaging examination; or (2) GFR <60 ml/(min·1.73 m^2^) ≥3 months, with or without renal injury. The CKD stage standard utilized was as follows: Stage 1: GFR ≥90 ml/(min·1.73 m^2^); Stage 2: GFR at 60–89 ml/(min·1.73 m^2^); Stage 3: GFR at 30–60 ml/(min·1.73 m^2^); Stage 4: GFR at 15–29 ml/(min·1.73 m^2^); and Stage 5: GFR <15 ml/(min·1.73 m^2^) or dialysis.

### 2.2. Diagnostic Criteria of Traditional Chinese Medicine

According to the guiding principles of clinical research on the treatment CRF with new Chinese medicine [9], the syndrome differentiation standard of damp-heat syndrome of traditional Chinese medicine was established. That is those who have three concurrent symptoms, such as little food intake, abdominal bloat, dry mouth, bitter mouth, nausea, vomiting, and/or yellow/greasy tongue coating.

### 2.3. Inclusion Criteria

The current experimental protocol received approval by the ethics committees of the experimental locations (approval number: 2012ah-040). The inclusion criteria were as follows: (1) aged between 18 and 70; (2) meeting CRF and damp-heat syndrome standard; (3) no peritoneal dialysis, hemodialysis, or kidney transplantation and a GFR <60 ml/(min·1.73 m^2^); (4) voluntarily participated in the clinical trial and signed the informed consent; and (5) early enough in the course of the disease to correct aggravating factors, such as hypertension, infection, calcium and phosphorus metabolism disorder, or acidosis.

### 2.4. Exclusion Criteria

The exclusion criteria were as follows: (1) severe heart or brain diseases that affect judgement of curative effects; (2) psychological disorders that prohibit cooperation; (3) pregnant or lactating women; (4) renal replacement therapy; (5) allergies to certain drugs in the study or participation in other clinical trials at the same time; and (6) patients prescribed immunosuppressants.

### 2.5. Clinical Data

A total of 316 patients with comorbid CRF and damp-heat syndrome were selected for participation from the First Affiliated Hospital of Anhui University of Traditional Chinese Medicine (the leading research unit of this project), Lu'an Hospital of Traditional Chinese Medicine, and Wuhu Hospital of Traditional Chinese Medicine. A total of 282 patients completed the full 12 weeks of treatment. There were no significant differences between the two groups in gender, age, or course of disease (*p* > 0.05). An additional 20 healthy individuals in our hospital were selected as a healthy comparison group. Experimental and control group information is shown in Table 1.

### 2.6. Study Groups

According to the treatment plan of comorbid CRF and damp-heat syndrome, participants were randomly divided into the experimental (routine Western medicine treatment + Qingshen granules) and control groups (routine Western medicine treatment only) at a 1:1 ratio, with the center (hospital) being the stratified factor. A total of 282 patients (136 in the experimental group and 146 in the control group) completed the 12-week course of treatment.

### 2.7. Treatment

Patients in the treatment and control groups were given routine Western medicine treatment, including dietary control, treatment of primary disease, and correction of acute aggravating factors. Specific attention was paid to the drug balance of the two groups to ensure that drugs that may have affected efficacy judgment were not used. In the experimental group, Qingshen granules (production batch number: 20110507) were added to the conventional treatment plan of Western medicine, which were composed of *Hedyotis diffusa*, rhubarb, coix seed, *Salvia miltiorrhiza*, *Atractylodes macrocephala*, plantain grass, and other drugs. Patients were given one bag of Qingshen granules three times per day. The course of treatment in both groups was 12 weeks.

### 2.8. Observation Indicators

Clinical disease effects of the experimental and control groups were measured by the content of CD4^+^ T cells, CD8^+^ T cells, Th17 cells, and nuclear factor-*κ*B (NF-*κ*B) p65 activity, which were all detected by flow cytometry. Additionally, serum levels of interleukin-17 (IL-17), interleukin-6 (IL-6), tumor necrosis factor-*α* (TNF-*α*), tumor necrosis factor receptor-associated factor 6 (TRAF6), fibronectin (FN), and type IV collagen (Col-IV) protein were measured by enzyme-linked immunosorbent assay (ELISA). IL-17, IL-6, FN, and Col-IV kits were purchased from Shanghai Xitang Biotechnology Co., Ltd. The production batch numbers were F01450, F01310, F00841, and F5704, respectively, and the detection ranges were 4.2 ng/L–1000 ng/L, 3.58 ng/L–300 ng/L, 8.6 ng/L–1000 ng/L, and 2.45 *µ*g/L–300 *µ*g/L, respectively. TNF-*α* and TRAF6 kits were purchased from Shanghai Xinyu Biotechnology Co., Ltd. The production batch numbers were xy-E10134 and xy-751Hu01, respectively, and the detection ranges were 7.8 ng/L–500 ng/L and 0.16 *µ*g/L–10 *µ*g/L, respectively. Isolated cell samples were incubated at room temperature for 15 min, with the appropriate amount of fluorescent group labeled antibody, washed twice with 1-2 ml PBS, centrifuged for 5 min, resuspended with 500 *µ*l PBS, and analyzed by flow cytometry.

### 2.9. Criteria for Efficacy Evaluation

The standard for evaluation of efficacy was derived according to the guiding principles of clinical research on the treatment of CRF with new Chinese medicine [9]. The clinical disease curative effects were rated as *effective* (syndrome score reduction ≥70%), *stable* (syndrome score reduced ≥30%, or *invalid* (clinical symptoms and signs according to traditional Chinese medicine demonstrate no obvious improvement or even aggravation, and the syndrome score has been reduced by less than 30%).

### 2.10. Statistical Analysis

SPSS 21.0 statistical software was used for data analysis, and an *χ*^2^ test was used for counting data. Data are expressed as $\bar{x}$ *±* *s*. The assumptions of normal distribution and homogeneity of variance were met. A *t*-test was used to determine differences; otherwise, nonparametric test was used. A *p* < 0.05 was considered to be statistically significant.

## 3. Results

### 3.1. Comparison of Clinical Disease Efficacy between the Two Groups

The total effective rate in the experimental group was 79.41%, which was better than the 67.12% observed in the control group. There was a significant difference between the two groups (*p* < 0.05). See Table 2.

### 3.2. Effect on Peripheral Blood CD4^+^ T Cells, CD8^+^ T Cells, Th17 Cells, and NF-*κ*B p65 Activity

Before treatment, there were no significant differences in CD4^+^/CD8^+^ T cell ratio, Th17 cell level, or NF-*κ*B p65 activity between the experimental and control groups (*p* > 0.05), both of which were significantly elevated when compared with the healthy comparison group (*p* < 0.05 or *p* < 0.01). After treatment, CD4^+^/CD8^+^ T cell ratio, Th17 cell level, and NF-*κ*B p65 activity in the experimental group were significantly lower than before treatment (*p* < 0.05 or *p* < 0.01). Conversely, NF-*κ*B p65 activity in the control group was significantly lower after treatment than before treatment (*p* < 0.01); however, the other indexes, while being lower after treatment than before treatment, failed to reach statistically significant differences (*p* > 0.05). Overall, the experimental group demonstrated a larger decrease than the control group (*p* < 0.05 or *p* < 0.01). See Table 3.

### 3.3. Effects on Serum Levels of IL-17, IL-6, TNF-*α*, TRAF6, FN, and Col-IV

Before treatment, there were no significant differences observed in the serum levels of IL-17, IL-6, TNF-*α*, TRAF6, FN, or Col-IV between the experimental and control groups (*p* > 0.05), although both groups were significantly higher than those observed in the healthy comparison group (*p* < 0.05 or *p* < 0.01). After treatment, levels of IL-17, IL-6, TNF-*α*, TRAF6, FN, and Col-IV in the experimental group were lower than those observed before treatment (*p* < 0.05 or *p* < 0.01). In the control group, levels of IL-17, TNF-*α*, TRAF6, FN, and Col-IV in the control group were significantly lower than those before treatment (*P* < 0.05 or *P* < 0.01); however, while the levels of IL-6 were lower after treatment than those observed before treatment, the difference did not reach statistical significance (*p* > 0.05). Overall, the decrease observed in the experimental group was larger than that observed in the control group (*p* < 0.05 or *p* < 0.01). See Table 4.

## 4. Discussion

Renal interstitial fibrosis (RIF) is the primary pathological basis of CRF. The production of various cytokines through immune dysfunction damages renal tubular epithelial cells and releases inflammatory mediators that aggravate the progression of RIF [10, 11]. Immune function in the body depends on the regulation of T cells, especially the mutual restriction and balance between CD4^+^ T cells and CD8^+^ T cells. The balance of the CD4^+^ T cell and CD8^+^ T cell ratio is a necessary condition for maintaining normal immune function in the body, with an imbalance of the ratio leading to immune dysfunction [12]. Th17 cells are produced by a specific subset of CD4^+^ T cells, and they are involved in the immune response through the secretion of inflammatory factors, such as IL-17, IL-6, and TNF-*α* [13]. The immune function of CRF patients is often severely disordered [14, 15], with the release of inflammatory mediators being involved in the course of CRF [4–6]. The high expression of inflammatory mediators can activate the NF-*κ*B signaling pathway of renal cells, inducing the release of a variety of inflammatory factors, which participate in the inflammatory response, extracellular matrix (ECM) deposition, and other pathological processes [16–19], ultimately promoting the formation of RIF.

In this study, the levels of peripheral blood CD4^+^ and CD8^+^ T cells, as well as the ratio of CD4^+^/CD8^+^ T cells, and the content of Th17 cells in CRF patients were significantly higher than those of healthy individuals. After treatment, the expression levels of the above indexes remained above normal values, suggesting that there was immune dysfunction in the CRF patients. Because of the immune dysfunction, CRF patients secrete too much IL-17 and IL-6. In turn, IL-6 promotes the proliferation and activation of Th17 cells, thus producing more IL-17 [20] and inducing renal tissue to produce a large amount of TRAF6 [21]. TRAF6 is an important regulator of the NF-*κ*B signaling pathway, and it is the only signal molecule in the TRAF family that directly binds to NF-*κ*B receptor activator, allowing it to activate NF-*κ*B signaling pathway [22]. In this study, NF-*κ*B p65 activity in the peripheral blood, as well as the expression of IL-17, IL-6, and TRAF6 in serum, was increased in CRF patients, suggesting that the immune dysfunction of CRF patients can promote a high expression of IL-17, IL-6, and TRAF6, thus activating NF-*κ*B signaling pathway and causing renal diseases. Activated NF-*κ*B can promote the secretion of TNF-*α*, a major downstream factor. TNF-*α* is an important mediator of early inflammatory response and an important pathogenic factor, and its expression gradually increases with the deterioration of renal function [23]. The absence of TRAF6 can significantly reduce the immune signal, NF-*κ*B activation, and downstream cytokines [24, 25], which also confirms the results of this study.

Activation of NF-*κ*B can also promote excessive secretion of adhesion molecules and chemokines, leading to ECM deposition and RIF. Overdeposition of ECM is one of the most important factors leading to RIF [26], which is mainly composed of collagen, FN, and other substances. Col- IV is the main collagen in the glomerular basement membrane, constructing its scaffold. It is secreted and produced by many kinds of cells in activated renal tissue, such as mesangial cells, endothelial cells, and renal tubular epithelial cells after being stimulated by inflammatory factors. Col-IV can stimulate its overexpression, leading to glomerulosclerosis and the production of RIF. Therefore, Col-IV is an important index reflecting RIF [27]. FN is a very important glycoprotein in ECM, which is primarily distributed in the tubulointerstitial area and connected by fibrous tissue to maintain the typical tissue structure of renal tubules. Under normal circumstances, FN is underexpressed in renal tissue. When stimulated by inflammatory factors, it can promote its overexpression and aggravate renal damage [28]. In this study, we found that the levels of serum Col-IV and FN protein in CRF patients were significantly increased, suggesting that ECM also significantly increased following inflammation, accelerating the formation of RIF. After the treatment of Qingshen granules, serum expression levels of Col-IV and FN protein in CRF patients significantly decreased, possibly reducing RIF progress.

Through clinical experience, Qingshen granules have become the primary prescription of our hospital for CRF patients. It is mainly composed of rhubarb, *Hedyotis, tuckahoe*, coix seed, *Salvia miltiorrhiza*, and other drugs. It has the effects of clearing heat and dampness, as well as removing blood stasis and turbidity. In the prescribed mixture, rhubarb, which can clear away heat, blood stasis, and turbidity, is the key component. Additionally, the mixture contains *Hedyotis*, wormwood, and *Coptis*, which are used to clear away heat and dampness; *Polyporus*, *Poria cocos*, *Atractylodes macrocephala*, coix seed, *Alisma orientale*, cardamom, lentil, and plantain, which are used to strengthen the spleen and reduce dampness; and motherwort and *Salvia miltiorrhiza,* which are used as adjuvants. The current results showed that the total effective rate of clinical diseases in the experimental group was 79.41%, which was superior to the 67.12% observed in the control group.

In conclusion, Qingshen granules, in combination with Western medicine, were found to significantly reduce NF-*κ*B p65 activity, as well as the expression of IL-17, IL-6, TNF-*α*, TRAF6, FN, and Col-IV in the peripheral blood of CRF patients. Further, it was found that the combination of treatment produced better clinical outcomes than Western medicine alone. It is indicated that Qingshen granules can significantly improve the clinical symptoms of patients and delay the progression of CRF and RIF, and their mechanism is related to effective regulation of immune dysfunction, inhibition of elevated expression of inflammatory mediators regulated by the TRAF6 activated NF-*κ*B pathway, and reduction of ECM deposition.

## Figures and Tables

**Table 1:** Comparison of general demographic data between the two groups.

Variable	Experimental group	Control group
Male/female	75/61	84/62
Age (year)	54.0 ± 10.5	51.8 ± 12.0
Duration (month)	27.6 ± 23.4	28.9 ± 22.9
Primary disease		
Chronic nephritis	74	79
Diabetic nephropathy	26	25
Hypertensive nephropathy	20	23
Polycystic kidney disease	6	8
Other	10	11
CKD stage		
CKD3	45	44
CKD4	41	47
CKD5	50	55

**Table 2:** Comparison of clinical disease efficacy between the two groups, *n* (%).

Groups	*n*	Effective	Stable	Invalid	Total efficacy (%)
Control group	146	42 (28.77%)	56 (38.35%)	48 (32.88%)	67.12
Experimental group	136	53 (38.97%)	55 (40.44%)	28 (20.59%)	79.41^*∗*^

*Note*. Compared with the control group, ^*∗*^*p* < 0.05.

**Table 3:** Comparison of peripheral blood levels of CD4^+^ T cells, CD8^+^ T cells, Th17 cells, and NF-*κ*B p65 activity.

Measure	Healthy comparison group (*n* = 20)	Control group (*n* = 146)		Experimental group (*n* = 136)	
		Before treatment	After treatment	Before treatment	After treatment
CD4^＋^ (%)	24.63 ± 6.89	58.25 ± 14.36^Δ^	48.34 ± 17.79	58.93 ± 14.83^Δ^	35.82 ± 13.94^#^*∗*^^
CD8^＋^ (%)	17.34 ± 6.34	29.35 ± 10.12^Δ^	26.64 ± 13.43	29.72 ± 10.35^Δ^	22.45 ± 10.83^#^*∗*^^
CD4^＋^/CD8^＋^	1.42 ± 0.61	1.96 ± 0.83^Δ^	1.81 ± 0.96	1.98 ± 0.86^Δ^	1.58 ± 0.72^#^*∗*^^
Th17 (%)	0.68 ± 0.25	2.46 ± 0.94^ΔΔ^	2.22 ± 1.17^##^	2.51 ± 1.05^ΔΔ^	1.70 ± 0.83^##^*∗∗*^^
NF-*κ*B p65 (%)	8.97 ± 2.96	36.45 ± 12.65^ΔΔ^	30.43 ± 14.05	36.84 ± 12.96^ΔΔ^	24.86 ± 1.97^#^

*Note*. Compared with the healthy comparison group, ^Δ^*p* < 0.05, ^ΔΔ^*p* < 0.01; compared with the same group before treatment,^#^*p* < 0.05, ^##^*p* < 0.01; and compared with the control group in the same period, ^*∗*^*p* < 0.05, ^*∗∗*^*p* < 0.01.

**Table 4:** Comparison of serum levels of IL-17, IL-6, TNF-*α*, TRAF6, FN, and Col-IV.

Measure	Healthy comparison group (*n* = 20)	Control group (*n* = 146)		Experimental group (*n* = 136)	
		Before treatment	After treatment	Before treatment	After treatment
IL-17 (ng/L)	12.48 ± 5.92	28.35 ± 13.21^Δ^	24.85 ± 15.32^#^	28.62 ± 13.53^Δ^	19.78 ± 12.25^#^*∗*^^
IL-6 (ng/L)	6.53 ± 1.26	76.55 ± 20.36^ΔΔ^	72.67 ± 21.48	77.13 ± 20.54^ΔΔ^	58.42 ± 18.25^#^*∗*^^
TNF-*α* (ng/L)	35.62 ± 14.18	109.25 ± 23.12^ΔΔ^	100.49 ± 28.48^##^	110.34 ± 23.76^ΔΔ^	75.49 ± 22.80^##^*∗∗*^^
TRAF6 (*μ*g/L)	2.74 ± 0.92	4.83 ± 1.65^Δ^	3.92 ± 1.99^##^	4.94 ± 1.82^Δ^	2.85 ± 1.53^##^*∗∗*^^
FN (ng/L)	24.86 ± 9.78	92.65 ± 20.62^ΔΔ^	87.46 ± 21.60^#^	93.42 ± 20.36^ΔΔ^	62.86 ± 19.35^##^*∗∗*^^
Col- IV (*μ*g/L)	11.34 ± 6.52	36.78 ± 14.25^ΔΔ^	32.65 ± 15.61^#^	36.85 ± 14.58^ΔΔ^	24.36 ± 13.36^##^*∗∗*^^

*Note*. Compared with the healthy comparison group, ^Δ^*p* < 0.05, ^ΔΔ^*p* < 0.01; compared with the same group before treatment ^#^*p* < 0.05, ^##^*p* < 0.01; and compared with the control group in the same period, ^*∗*^*p* < 0.05, ^*∗∗*^*p* < 0.01.

## Data Availability

Readers can access the data underlying the findings of this study by contacting the corresponding author's email address (wypwyp54@aliyun.com) or on the Chinese Clinical Trial Registry (ChiCTR-INR17011057).
